# Having concomitant asthma phenotypes is common and independently relates to poor lung function in NHANES 2007–2012

**DOI:** 10.1186/s13601-018-0201-3

**Published:** 2018-05-04

**Authors:** Rita Amaral, João A. Fonseca, Tiago Jacinto, Ana M. Pereira, Andrei Malinovschi, Christer Janson, Kjell Alving

**Affiliations:** 1 CINTESIS- Center for Health Technology and Services Research, Faculty of Medicine, University of Porto, Edifício Nascente, Piso 2, Rua Dr. Plácido da Costa, s/n, 4200-450 Porto, Portugal; 2 Department of Cardiovascular and Respiratory Sciences, Porto Health School, Porto, Portugal; 3 MEDCIDS- Department of Community Medicine, Information, and Health Sciences, Faculty of Medicine, University of Porto, Porto, Portugal; 4 Department of Allergy, Instituto & Hospital CUF, Porto, Portugal; 5 Department of Medical Sciences: Clinical Physiology, Uppsala University, Uppsala, Sweden; 6 Department of Medical Sciences: Respiratory Medicine and Allergology, Uppsala University, Uppsala, Sweden; 7 Department of Women’s and Children’s Health: Paediatric Research, Uppsala University, Uppsala, Sweden

**Keywords:** Asthma, Asthma-related outcomes, Epidemiological study, Overlap, Phenotypes

## Abstract

**Background:**

Evidence for distinct asthma phenotypes and their overlap is becoming increasingly relevant to identify personalized and targeted therapeutic strategies. In this study, we aimed to describe the overlap of five commonly reported asthma phenotypes in US adults with current asthma and assess its association with asthma outcomes.

**Methods:**

Data from the National Health and Nutrition Examination Surveys (NHANES) 2007–2012 were used (n = 30,442). Adults with current asthma were selected. Asthma phenotypes were: B-Eos-high [if blood eosinophils (B-Eos) ≥ 300/mm^3^]; FeNO-high (FeNO ≥ 35 ppb); B-Eos&FeNO-low (B-Eos < 150/mm^3^ and FeNO < 20 ppb); asthma with obesity (AwObesity) (BMI ≥ 30 kg/m^2^); and asthma with concurrent COPD. Data were weighted for the US population and analyses were stratified by age (< 40 and ≥ 40 years old).

**Results:**

Of the 18,619 adults included, 1059 (5.6% [95% CI 5.1–5.9]) had current asthma. A substantial overlap was observed both in subjects aged < 40 years (44%) and ≥ 40 years (54%). The more prevalent specific overlaps in both age groups were AwObesity associated with either B-Eos-high (15 and 12%, respectively) or B-Eos&FeNO-low asthma (13 and 11%, respectively). About 14% of the current asthma patients were “non-classified”. Regardless of phenotype classification, having concomitant phenotypes was significantly associated with (adjusted OR, 95% CI) ≥ 2 controller medications (2.03, 1.16–3.57), and FEV_1_ < LLN (3.21, 1.74–5.94), adjusted for confounding variables.

**Conclusions:**

A prevalent overlap of commonly reported asthma phenotypes was observed among asthma patients from the general population, with implications for objective asthma outcomes. A broader approach may be required to better characterize asthma patients and prevent poor asthma outcomes.

**Electronic supplementary material:**

The online version of this article (10.1186/s13601-018-0201-3) contains supplementary material, which is available to authorized users.

## Background

Profiling asthma phenotypes is becoming increasingly relevant to choose the most appropriate therapeutic strategy for individual patients, and to provide optimal improvement of disease control and quality of life [1, 2].

The predominant pathophysiological mechanism of asthma is type 2-mediated, associated with atopy and eosinophilic inflammation [3, 4]. However, it has been shown that asthma is a heterogeneous disease that involves other mechanisms that are not so well understood and respond poorly to corticosteroid therapy (e.g. non-type 2-mediated) [3, 5, 6].

There has been a recent rise in the number of studies that try to identify asthma phenotypes based on non-invasive type 2-markers, such as blood eosinophils (B-Eos) count, fraction of exhaled nitric oxide (FeNO), serum IgE, and/or serum periostin [7–10]. Moreover, there appears to be an additive role of biomarkers, such as B-Eos and FeNO, in relation to recent asthma morbidity [7, 11, 12].

However, there is little information regarding the appropriate use of these biomarkers in asthma phenotype classification, particularly when a significant overlap occurs. Also, the importance of having concomitant asthma phenotypes for disease outcomes has scarcely been studied in the general population. This information may be useful to identify personalized and targeted therapeutic strategies [13, 14].

Recently, an extensive overlap of asthma phenotypes was described [15]. However, only type 2-high, atopic, and eosinophilic asthma were examined. The extent of overlap with other phenotypes commonly reported in the literature, among adults with asthma from the general population remains unknown. Asthma phenotypes are frequently reported in the literature according to the high levels of systemic and local type 2-markers (B-Eos high and FeNO-high, respectively) [1–4, 16–18]. However, other distinct subgroups of asthma phenotypes are increasingly being reported due to its characteristics of steroid therapy resistance and lack of inflammatory markers: e.g. subjects with asthma without evidence of type 2 inflammation (Th2-low phenotype); obese asthmatic subjects (obesity-related asthma phenotype); and patients with asthma-COPD overlap syndrome [19–22].Therefore, we hypothesized that if, in general population, occurs a high proportion of overlap of commonly reported asthma phenotypes, there may be a need for improving the definition of asthma phenotypes. Additionally, asthma subjects with multiple phenotypes may have poorer asthma-related outcomes.

The aims of this study were to describe the proportion of overlap of five commonly reported asthma phenotypes: asthma with obesity (AwObesity), asthma with concurrent COPD (AwCOPD), B-Eos-high, FeNO-high and B-Eos&FeNO-low asthma, and to examine the association of their overlap with asthma-related outcomes, using population-based data from the National Health and Nutrition Examination Surveys (NHANES), 2007–2012.

## Methods

### Study design

The NHANES is a nationally representative survey of the civilian, non-institutionalized U.S. population that uses a complex stratified, multistage probability sampling. Further details on NHANES survey design databases can be found in Additional file 1: Supplementary methods. The National Center for Health Statistics, Ethics Review Board approved NHANES protocol, and all participants gave written informed consent.

### Subjects selection

**Fig. 1:**
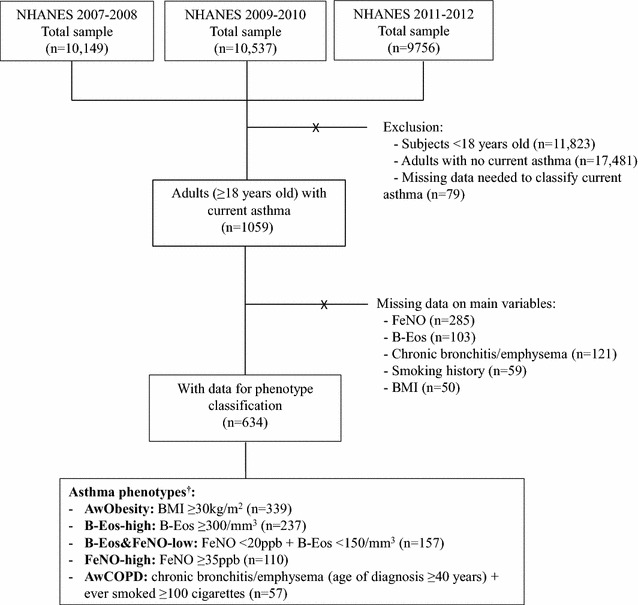
Flowchart of the study analysis. ^†^Seventy-seven patients were considered “non-classified” (non-single and non-multiple phenotype)

Six survey years (NHANES 2007–2012) were analyzed, resulting in 30,442 individuals of all ages (Fig. 1). We included adults (≥ 18 years-old) with current asthma (n = 1059), defined by a positive answer to the questions: “Has a doctor ever told you that you have asthma?” together with “Do you still have asthma?”, and either “wheezing/whistling in the chest in the past 12 months” or “asthma attack in the past 12 months.”

### Variables

Demographic characteristics, such as age, gender, body mass index (BMI), race/ethnicity, and educational status were analyzed. B-Eos count, FeNO and spirometric measurements, collected at the NHANES Mobile Examination Center were also examined. A detailed description of the procedures can be found elsewhere [23–25]. FeNO and spirometric measurements not fulfilling ATS/ERS recommendations [26, 27] were excluded (n = 653). After predicted values of basal FEV_1_ and FEV_1_/FVC were calculated [28], with a correction factor for ethnicity [29], abnormal lung function was defined if either one of them were less than the lower limit of normal (LLN), defined as lower fifth percentile of the reference population [30].

Prescription medications used last month were also analyzed [31]. More details regarding the inclusion of reliever and controller medications for asthma and the definitions of each asthma-related variable included in the analysis (asthma attack, asthma-related emergency department (ED) visit, work/school absenteeism, asthma symptoms, smoking status and rhinitis) are provided in the supplementary material (see Additional file 1: Supplementary methods).

### Asthma phenotypes definition

A B-Eos count ≥ 300/mm^3^ was used to define an B-Eos-high asthma phenotype [32, 33], while FeNO-high was defined as FeNO ≥ 35 ppb [34]. Asthma patients with both B-Eos < 150/mm^3^ and FeNO < 20 ppb were categorized as B-Eos&FeNO-low asthma [35]. Additionally, we considered subjects with either B-Eos-high or FeNO-high as having “Type 2-high” asthma.

The AwObesity phenotype was defined by a BMI ≥ 30 kg/m^2^ in individuals with current asthma [36]. Finally, the AwCOPD phenotype was considered if participants ≥ 40 years-old had concurrent asthma and COPD, defined by a positive answer to “Has a doctor ever told you that you have chronic bronchitis/emphysema”, with age of diagnosis ≥ 40 years and having self-reported smoking history (being either a current or ex-smoker) [37, 38].

### Statistical analysis

In accordance with the NHANES sampling design, the weights for each full sample 2-year mobile examination center were used to obtain weighted percentages adjusted to the US adult population.

Categorical variables were described as frequencies and weighted proportions, and continuous variables were described as median and first and third quartiles (Q1–Q3). Chi square test and Mann–Whitney U-test were used to compare groups. To explore the association of concomitant (having at least 2 concurrent) phenotypes with each asthma-related outcome we performed multivariate logistic regression modelling. Separate models were run using each asthma-related outcome and abnormal lung function as dependent variable and having multiple phenotypes as independent variables. Adjustments were also made for potential confounders: sex, age, race, current smoking and rhinitis. Adjusted odds ratios (aOR) with 95% confidence intervals (95% CI) were presented, and model fit was assessed using the *svylogitgof* function [39].

According to age (< 40 or ≥ 40 years-old), a four- or five-set Venn-Euler diagram was used to quantify the proportion of individuals with different asthma phenotypes and to illustrate the overlap.

The diagrams were created using R software version 3.2.0 (“VennDiagram”, “venneuler” and “reshape2” packages) and all statistical analyses were performed in Stata version 13.1 (StataCorp, TX, USA), using the *survey* command to account for the complex sampling design and weights in the NHANES. The *MI* command was used to perform sensitivity analysis by multiple-imputation of missing values; however, to create the Venn-Euler diagrams, a listwise deletion for missing data was applied. A *p* value < 0.05 was considered statistically significant.

## Results

Of the 18,619 adults included in NHANES 2007–2012 datasets, 1059 (5.6% [95% CI 5.1–5.9]) had current asthma (Fig. 1). Of these, 63% were female, and the median (Q1–Q3) age was 48.0 (32.0–62.0) years. After excluding subjects with missing data on the main variables, 634 individuals were included for phenotype classification (Fig. 1). Despite having all information available, 77 patients did not meet the criteria for any of the defined asthma phenotypes and were considered “non-classified”. These were non-obese subjects with asthma who did not meet the criteria for COPD, had B-Eos values ranging between 150 and 300/mm^3^, and FeNO ranging 20–34 ppb.

Demographic characteristics of adults with current asthma included and excluded from the analysis and patients with single (n = 271) and multiple phenotypes (n = 286) are described in Table 1.

**Table 1:** Characteristics of adults with current asthma: included and excluded from phenotype classification, and stratified by single or multiple phenotypes

Demographic characteristics n (wt%)	Included subjects n = 634	Excluded subjects n = 425	*p* value*	Single phenotype^†^ n = 271	Multiple phenotypes^†^ n = 286	*p* value*
Female gender	410 (63)	261 (64)	0.93	174 (64)	192 (66)	0.68
Age (yrs), median (Q1–Q3)	44.0 (31.0–57.0)	48.9 (33.7–68.0)	*<* *0.001*	42.0 (30.0–55.0)	47.5 (34.0–60.0)	*0.003*
BMI (kg/m^2^), median (Q1–Q3)	30.8 (25.4-35.9)	31.4 (24.4-35.7)	0.99	28.7 (24.2–35.0)	33.7 (30.7–39.0)	*<* *0.001*
Obesity status						
Underweight (≤ 18.4 kg/m^2^)	2 (0.3)	13 (4)	< *0.001*	1 (0.2)	1 (0.5)	0.41
Normal (18.5–24.9 kg/m^2^)	142 (25)	98 (27)	0.44	78 (30)	20 (8)	< *0.001*
Overweigh (25–29.9 kg/m^2^)	151 (26)	77 (19)	0.07	80 (32)	38 (15)	< *0.001*
Obese (≥ 30 kg/m^2^)	339 (49)	187 (49)	0.94	112 (38)	227 (77)	< *0.001*
Race and/or ethnicity						
Hispanic	105 (8)	85 (10)	0.30	38 (8)	56 (9)	0.24
Non-Hispanic white	323 (74)	184 (63)	*0.005*	135 (72)	138 (72)	0.93
Non-Hispanic black	167 (14)	117 (18)	*0.03*	76 (14)	81 (16)	0.71
Other Race	39 (4)	39 (7)	0.06	22 (6)	11 (3)	0.11
Smoking status						
Current smoker	199 (29)	114 (32)	0.40	89 (31)	87 (27)	0.46
Ex-smoker	163 (29)	98 (23)	0.13	56 (20)	89 (36)	*0.003*
Non-smoker	272 (43)	154 (45)	0.53	126 (49)	110 (37)	*0.02*
Education						
≥ High school	478 (84)	225 (69)	< *0.001*	205 (86)	209 (81)	0.13
Asthma-related medication^‡^						
Reliever medication**	276 (41)	202 (48)	0.16	113 (37)	132 (47)	0.10
Oral corticosteroids	33 (8)	29 (3)	*0.001*	12 (3)	15 (4)	0.75
Inhaled corticosteroids^§^	153 (25)	122 (30)	0.14	55 (19)	81 (32)	*0.01*
Other control medications^||^	53 (9)	63 (14)	0.19	15 (8)	33 (10)	0.50
Asthma phenotype						
AwObesity	339 (49)	–		112 (38)	227 (76)	< *0.001*
B-Eos-high	237 (36)	–		61 (22)	176 (62)	< *0.001*
B-Eos&FeNO-low	157 (26)	–		74 (30)	83 (30)	0.95
FeNO-high	110 (18)	–		18 (8)	92 (34)	< *0.001*
AwCOPD	57 (8)	–		6 (2)	51 (17)	< *0.001*
Non-classified^††^	77 (14)	–		–	–	

Data presented as absolute numbers and proportions weighted for the U.S. population. *p* values < 0.05 are presented in italic

*Yrs* years, *BMI* body mass index, *Q1* first quartile, *Q3* third quartile, *BMI* body mass index, *AwObesity* Asthma with obesity, *AwCOPD* Asthma with concurrent COPD

* Chi square test or Mann–Whitney U-test was used

^†^Seventy seven subjects included in the “non-classified” group were considered as missing

^‡^Prescribed medication taken in the past 30 days

^§^Alone or in combination with long-acting inhaled β_2_-agonist

^||^Included long-acting inhaled β_2_-agonist (without corticosteroids), leukotriene inhibitors, and mast cell stabilizers

**Short-acting β_2_-agonist and/or anticholinergic

^††^Subjects with non-single and non-multiple asthma phenotype

There is a female predominance in both groups (64 and 66%, respectively). Subjects with multiple phenotypes were older (*p* = 0.003), had higher BMI (*p* < 0.001), were more often obese (*p* < 0.001) and ex-smokers (*p* = 0.003), and a higher proportion of patients were treated with inhaled corticosteroids (ICS) (*p* = 0.01), than those with only one phenotype. Females were more obese, regardless the number of concomitant asthma phenotypes (data not shown).

### Phenotypes and overlap description

The weighted proportions of asthma phenotypes were (in descending order): 49% for AwObesity, 36% for B-Eos-high asthma, 26% for B-Eos&FeNO-low asthma, 18% for FeNO-high asthma, and 8% for AwCOPD (Table 1).

Demographic and clinical characteristics among all 5 asthma phenotypes and the “non-classified” group are described in Table 2.

**Table 2:** Demographic and clinical characteristics among all 5 phenotypes and in the “Non-classified” group

Characteristics n (wt%)	AwObesity n = 339	B-Eos-high n = 237	B-Eos&FeNO-low n = 157	FeNO-high n = 110	AwCOPD n = 57	Non-classified^††^ n = 77
Female gender	366 (67)	196 (55)	138 (78)	78 (52)	71 (58)	44 (56)
Age (yrs), median (Q1–Q3)	48.0 (34.0–59.0)	47.0 (31.0–59.0)	41.0 (27.0–57.0)	45.0 (30.0–54.0)	61.0 (52.0–69.0)	39.0 (28.0–53.0)
BMI (kg/m^2^), median (Q1–Q3)	35.4 (32.5–40.4)	30.3 (25.9–36.8)	28.9 (24.2–33.0)	27.6 (24.9–33.4)	30.3 (25.1–35.1)	24.3 (22.8–27.5)
Race and/or ethnicity						
Hispanic	94 (9)	78 (11)	30 (8)	29 (10)	18 (4)	11 (6)
Non-Hispanic white	230 (65)	175 (73)	79 (69)	65 (75)	80 (81)	50 (86)
Non-Hispanic black	177 (21)	74 (12)	62 (17)	38 (13)	27 (11)	10 (6)
Other race	25 (5)	21 (4)	13 (6)	6 (2)	10 (4)	6 (2)
Smoking status						
Current smoker	150 (27)	104 (32)	48 (23)	18 (13)	65 (52)	23 (28)
Ex-smoker	134 (27)	100 (32)	35 (25)	42 (39)	70 (48)	18 (30)
Non-smoker	229 (45)	124 (35)	88 (51)	66 (49)	0 (0)	36 (42)
Education						
≥ High school	358 (79)	227 (80)	125 (81)	102 (87)	73 (59)	64 (91)
Asthma-related medication^‡^						
Reliever medication**	234 (42)	172 (51)	82 (44)	65 (46)	69 (57)	31 (36)
Oral corticosteroids	36 (5)	17 (5)	4 (2)	10 (7)	15 (7)	2 (2)
Inhaled corticosteroids^§^	144 (27)	103 (32)	36 (20)	36 (33)	60 (47)	17 (19)
Asthma-related outcomes						
Asthma attack	363 (68)	252 (74)	125 (68)	98 (71)	85 (63)	54 (75)
Asthma-related ED	130 (27)	80 (23)	41 (23)	26 (13)	37 (32)	9 (8)
> 2 asthma symptoms	311 (66)	199 (65)	92 (55)	80 (57)	90 (74)	42 (59)
Work/school absenteeism	66 (18)	43 (16)	25 (18)	23 (14)	12 (20)	10 (14)
Lung function						
FEV_1_% predicted, median (Q1–Q3)	89.0 (75.6–99.2)	84.1 (75.2–95.4)	93.2 (83.8–100.8)	82.7 (75.9–95.4)	74.0 (62.9–90.1)	89.9 (80.5–103.5)
FEV_1_/FVC, median (Q1–Q3)	0.77 (0.62–0.82)	0.74 (0.65–0.80)	0.79 (0.72–0.83)	0.72 (0.66–0.79)	0.63 (0.50–0.75)	0.76 (0.69–0.82)

Data presented as absolute numbers and proportions weighted for the U.S. population

*Yrs* years, *BMI* body mass index, *Q1* first quartile, *Q3* third quartile, *BMI* body mass index, *AwObesity* Asthma with obesity, *AwCOPD* Asthma with concurrent (COPD), *ED* emergency-department, *FEV1* Forced expiratory volume in 1 s, *FEV1/FVC* forced expiratory volume in 1 s and functional vital capacity ratio, *LLN* lower limit of normality

^††^Subjects with non-single and non-multiple asthma phenotype

^‡^Prescribed medication taken in the past 30 days

** Short-acting β_2_-agonist and/or anticholinergic

^§^Alone or in combination with long-acting inhaled β_2_-agonist

There is a female predominance among all phenotypes, particularly in the B-Eos&FeNO-low (78%). Subjects with AwCOPD phenotype were the oldest group (median [Q1–Q3]: 61.0 [52.0–69.0] years-old), with the lowest proportion of individuals that had ≥ high school and lowest FEV_1_/FVC (0.63 [0.50–0.75]), comparing to the other phenotypes.

**Fig. 2:**
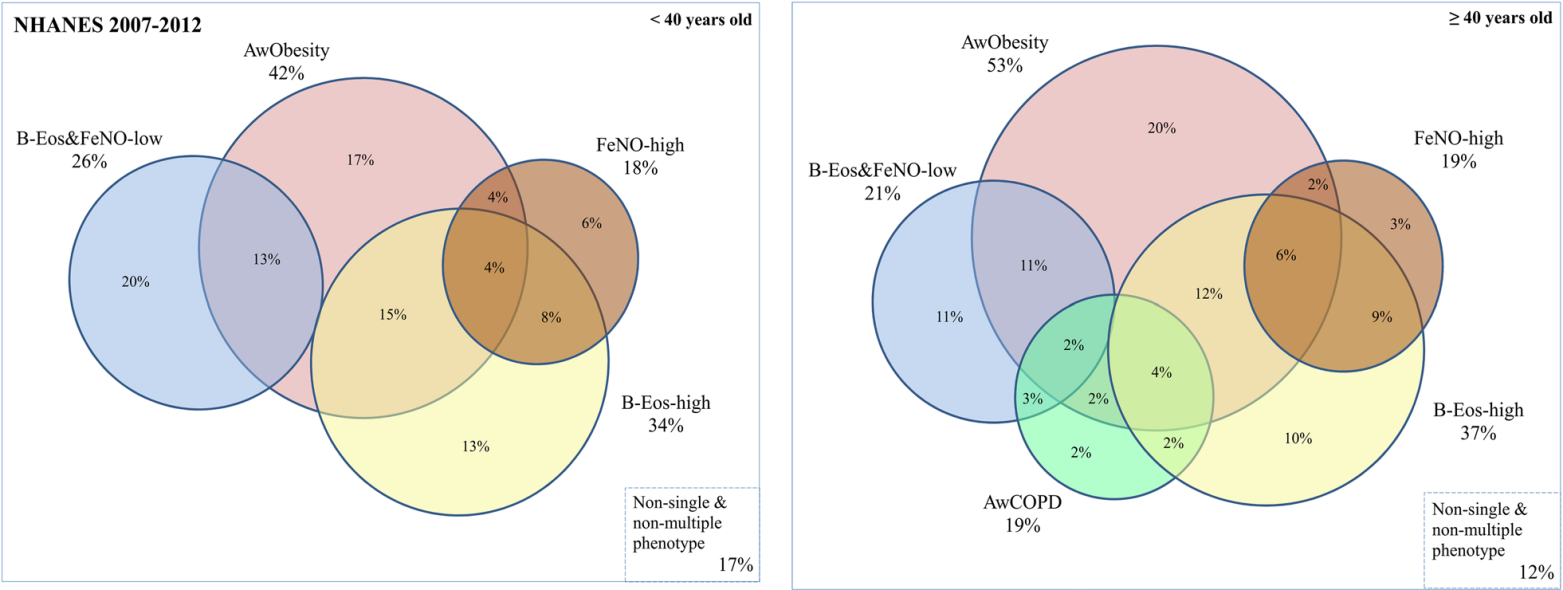
Venn-Euler diagrams quantifying the overlap among the asthma phenotypes, stratified by age. Data presented in weighted percentages to the US population. The overlap between AwCOPD with FeNO-high asthma (0.1%) and the overlap between AwCOPD, FeNO-high, AwObesity and B-Eos-high (0.5%) phenotypes are not shown

When categorized by age, < 40 (n = 227) and ≥ 40 years-old (n = 330), the most prevalent phenotypes were AwObesity (42 and 53%, respectively) and B-Eos-high asthma (34 and 37%). The less ones were FeNO-high asthma (18 and 19%) and AwCOPD (19% in the older group) (Fig. 2).

The areas of intersection in the four- and five-set Venn-Euler diagrams revealed 5 and 12 overlapping categories, and proportions of 17 and 12% of non-classified asthma subjects, respectively.

In both diagrams, a substantial total overlap was observed: 44% in subjects < 40 years-old and 54% in subjects ≥ 40 years-old. About 40% of the individuals in both age groups had two concomitant asthma phenotypes, 4% of the younger group had 3 concomitant phenotypes and 13% of the older group had ≥ 3 (Table 3 and Fig. 2). Furthermore, 1% of the older subjects had four concomitant asthma phenotypes: AwObesity, AwCOPD, FeNO-high, and B-Eos-high asthma.

**Table 3:** Distribution and comparisons of the asthma-related outcomes among asthma phenotypes, stratified by age

	Total n (wt%)	Asthma attack	Asthma-related ED	≥ 2 asthma symptoms	Work/school absenteeism	Asthma medication		Lung function		
						≥ 1 reliever medication^†^	≥ 2 controller medication	FEV_1_ < LLN	FEV_1_% predicted^§^	FEV_1_/FVC < LLN
< 40 yrs										
1 phenotype	118 (56)	85 (70)	27 (20)	59 (56)	15 (21)	55 (43)	17 (11)	10 (7)	95.6 (90.2–102.1)	26 (20)
2 phenotypes	97 (40)	69 (75)	26 (32)	57 (73)	12 (9)	41 (40)	8 (9)	19 (23)	91.8 (81.3–99.2)	25 (29)
3 phenotypes	12 (4)	10 (73)	5 (40)	8 (72)	4 (25)	9 (68)	1 (4)	3 (36)	81.9 (75.3–84.6)	4 (29)
*p* value*										
1 versus 2		0.43	0.10	0.052	*0.04*	0.72	0.64	*0.006*	0.07	0.25
2 versus 3		0.92	0.55	0.96	0.07	0.15	0.46	0.43	*0.009*	0.98
1 versus 3		0.86	0.11	0.39	0.73	0.20	0.34	*0.01*	< *0.001*	0.48
≥ 40 yrs										
1 phenotype	153 (46)	104 (65)	35 (23)	76 (62)	20 (15)	58 (33)	26 (17)	29 (20)	91.2 (81.6–99.2)	34 (26)
2 phenotypes	136 (41)	92 (69)	28 (21)	70 (55)	12 (9)	60 (46)	43 (36)	31 (37)	80.4 (70.0–91.7)	27 (32)
≥ 3 phenotypes	41 (13)	26 (72)	4 (9)	30 (70)	8 (40)	22 (60)	16 (40)	12 (46)	74.0 (63.0–85.8)	16 (53)
*p* value*										
1 versus 2		0.56	0.82	0.38	0.41	0.12	*0.02*	*0.01*	*0.007*	0.46
2 versus ≥ 3		0.80	0.27	0.30	*0.002*	0.24	0.67	0.47	0.22	0.09
1 versus ≥ 3		0.50	0.21	0.54	*0.03*	0.053	*0.02*	*0.01*	*0.006*	*0.02*

Data presented as absolute numbers and proportions weighted for the U.S. population. *p* values < 0.05 are presented in italic. The 77 subjects included in the “non-classified” group were considered as missing

*ED* emergency-department, *FEV*_*1*_ forced expiratory volume in 1 s, *FEV*_*1*_*/FVC* forced expiratory volume in 1 s and functional vital capacity ratio, *LLN* lower limit of normality, *Q1* first quartile; Q3: third quartile

* Chi square test or Mann–Whitney U-test was used

^†^Short-acting β_2_-agonist or/and anticholinergic

^§^Presented as median (Q1–Q3)

The most prevalent overlaps in both groups (< 40 and ≥ 40 years-old) were AwObesity together with either B-Eos-high (15 and 12%, respectively) or B-Eos&FeNO-low asthma (13 and 11%) (Fig. 2).

Moreover, the proportions of subjects having AwObesity together with other phenotypes were high: 53% for the B-Eos-high phenotype, 48% for AwCOPD, 45% for the B-Eos&FeNO-low, and 44% for the FeNO-high phenotype. Also, the proportion of individuals having AwCOPD together with the B-Eos-high phenotype was high (36%), whereas the proportions were lower for the B-Eos&FeNO-low and the FeNO-high asthma phenotypes (15 and 10%, respectively) (data not shown).

In this population, only 12 and 15% of asthma subjects (< 40 and ≥ 40 years-old, respectively) with high B-Eos count had a concomitant high FeNO values (Fig. 2). Moreover, the two biomarkers were non-congruent across cut-offs. For example, when comparing groups with B-Eos count < 150/mm^3^ and 150–300/mm^3^, the proportion of asthma subjects having low FeNO (< 20 ppb), was not significantly different (Additional file 2: Table S1).

### Associations between asthma-related outcomes and phenotype overlap

A comparison of the clinical characteristics of participants with one, two or three or more asthma phenotypes, stratified by age, is presented in Table 3 and no significant differences were observed in any age groups with regard to asthma attacks, asthma-related ED, ≥ 2 asthma symptoms, and use of ≥ 1 reliever. In the older group, the proportion of individuals with work/school absenteeism, ≥ 2 controller medications and with FEV_1_/FVC < LLN was significantly higher in participants with concomitant phenotypes than in those with a single phenotype (Table 3). In both age groups, the proportion of patients with FEV_1_ < LLN was significantly higher when participants presented multiple phenotypes, as well as they presented lower median FEV_1_% predicted values.

When analyzing the asthma-related outcomes in subjects with a single phenotype with those having specific combination of asthma phenotypes, the overall findings were that subjects having multiple phenotypes had significantly higher proportion of using ≥ 1 reliever and ≥ 2 controller medications and had decreased lung function, with the exception of those with the B-Eos&FeNO-low phenotype combined with any of the other phenotypes (Additional file 3: Table S2, and Additional file 4: Table S3).

Moreover, a lower proportion of subjects reporting asthma attacks was observed in subjects with AwObesity and either FeNO-high (26%) or AwCOPD (20%), compared to those with a single phenotype (67%) (Additional file 3: Table S2). Subjects with concomitant AwCOPD and B-Eos&FeNO-low phenotypes had the lowest proportion of ≥ 2 asthma symptoms (20%), but had the highest proportion of using ≥ 1 reliever medication (84%) as well as having FEV_1_ < LLN (71%).

In multivariate regression analysis, adjusting for co-variables, having multiple phenotypes was significantly associated with using ≥ 2 controller medications (aOR, 95% CI 2.03, 1.16–3.57), and having reduced FEV_1_ (3.21, 1.73–5.94) (Table 4). However, no associations were seen with asthma attacks, asthma-related ED, ≥ 2 asthma symptoms, work/school absenteeism, use of reliever medication or FEV_1_/FVC < LLN (Additional file 5: Table S4).

**Table 4:** Regression models with significant associations between having multiple asthma phenotypes and asthma-related outcomes, adjusted for co-variates

	≥ 2 controller medications		FEV_1_ < LLN	
	aOR	95% CI	aOR	95% CI
Multiple versus single phenotype	*2.03*	*1.16–3.57*	*3.21*	*1.74–5.94*
Female gender	1.39	0.77–2.50	1.51	0.81–2.81
Age ≥ 40 yrs	*3.01*	*1.52–5.95*	*2.55*	*1.29–5.03*
Caucasian versus others	1.38	0.86–2.23	1.37	0.78–2.42
Current smoker versus non-/ex-smokers	1.02	0.52–2.02	*2.01*	*1.21–3.33*
Rhinitis	1.08	0.57–2.16	0.94	0.54–1.63
Goodness-of-fit test				
χ^2^ (*p* value)	0.86 (0.56)		0.80 (0.61)	

Multivariate logistic regression models adjusted for gender, age, race, current smoking and rhinitis. The aOR values with *p* < 0.05 are presented in italic

*FEV*_*1*_ forced expiratory volume in 1 s, *LLN* lower limit of normal, *CI* confidence interval, *aOR* adjusted odds ratio, *χ*^*2*^ Chi square goodness-of-fit

Furthermore, subjects aged ≥ 40 years-old, had significantly higher odds of using ≥ 2 controller medications and having FEV_1_ < LLN predicted, compared to those < 40 years-old, adjusted for covariates (Table 4). Being a current smoker was significantly associated with using ≥ 1 reliever medication (1.95, 1.35–2.83) and with reduced lung function: FEV_1_ < LLN predicted (2.01, 1.21–3.33) and FEV_1_/FVC < LLN (2.02, 1.16–3.51) and not associated with any other asthma-related outcomes (Table 4 and Additional file 5: Table S4).

The association between having concomitant phenotypes and using multiple controller medications was consistent when considering oral corticosteroids (OCS) separated from other controller medications (1.87, 1.09–3.21) (data not shown).

We also analyzed the potential bias of controller medications in the phenotype classification, particularly in the B-Eos-high and FeNO-high phenotypes (Additional file 6: Fig. S1). No significant differences in asthma-related treatment were found between the phenotypes, with exception for a higher proportion of patients treated with ICS within the FeNO-high and B-Eos-high phenotypes compared to those with B-Eos&FeNO-low phenotype (*p* = 0.03). When restricting to subjects with a single asthma phenotype no significant differences were found.

Moreover, sensitivity analyses showed that the proportion of total overlap (weighted 53%), and the associations between having multiple phenotypes and asthma outcomes were similar when imputing all missing values (data not shown). The goodness-of-fit test revealed adequate fitting for all regression models, except when using FEV_1_/FVC < LLN as dependent variable (Additional file 5: Table S4) and no statistically significant interactions between co-variables were observed.

## Discussion

We report a substantial overlap of commonly reported asthma phenotypes among adults with current asthma in a large population sample, with almost half of them having two or more concomitant phenotypes. Furthermore, having multiple asthma phenotypes, regardless of their classification, was associated with poorer asthma outcomes, particularly the use of more controller medication and reduced lung function.

These findings illustrate the complexity and unique features of the concomitant asthma phenotypes when categorizing asthma in adults, using only the “classical” (hypothesis-driven) approach, based on measures readily available in the clinic (such as non-invasive biomarkers and medical records).

Hypothesis-driven asthma phenotypes are usually based on single dimensions of the disease, such as clinical symptoms, triggers, pathology or patterns of airway obstruction [16–18, 40–42]. However, evidence has shown that this approach is highly heterogenous, as it depends on the a priori assumptions and target population [43–45]. Also, it is of note that the 77 subjects with asthma that could not be classified as having any of the studied phenotypes, supports the fact that there is a considerable number of asthma patients whose clinical phenotype is not easily classified (e.g. asthmatics with irreversible airflow obstruction, patients with similar airways symptoms but with different pattern of airway inflammation), suggesting the presence of sub-phenotypes [1, 22, 44, 45].

In an attempt to explore the pathophysiology of specific asthma subgroups, and help stratify patients for targeted therapies, data-driven or unsupervised approaches (such as k-means, hierarchical clustering, partition-around-medoids methods or latent class analysis) are being applied in airways disease to identify “novel” accurate and distinct phenotypes, taking into account the heterogeneity and multidimensional characteristics of the disease [8, 46–52].

Our study results seem to be in line with the view of those that argue for a combination of both hypothesis- and data-driven approaches as a way forward to progress our knowledge on asthma endotypes and clinical phenotypes in an iterative way [52–54]. The data-driven phenotypes studies obtained some of the phenotypes already defined by hypothesis-driven approaches (e.g. obese, non-eosinophilic asthmatics [8]; persistent airway inflammation [46]; low type-2 inflammation [49]; fixed obstructive, non-eosinophilic and neutrophilic [50]), but, importantly, they identified other phenotypes that differ by certain characteristics: clinical parameters [8, 47–49], clinical response to treatment [46, 52], and airway inflammation [49, 51]. Therefore, further studies are required to compare and validate the asthma phenotypes obtained using different unsupervised methods.

The high overlap of asthma phenotypes seen in this study was similar to the findings of Tran et al. [15], who used datasets from previous NHANES surveys to evaluate the overlap of asthma phenotypes. However, Tran et al. study focused on allergic asthma phenotypes, based on IgE levels, and was therefore limited to the 2005–2006 survey that lacks data on FeNO. We provided a broader analysis of phenotypes that included not only the eosinophil-based phenotype (associated more closely with IL-5-driven) and the one based on FeNO values (mostly dependent on IL-4/IL-13-driven) [12], but also other phenotypes not defined by biomarkers and in a much larger dataset.

In this study, we extended previous observations [7, 11] suggesting that FeNO and B-Eos count partially reflect different inflammatory pathways, representing a local and a systemic type 2-marker, respectively. We observed that only 12–15% of asthma subjects with high B-Eos count had concomitant high FeNO levels, in this population. Also, a similar proportion of subjects with multiple phenotypes was obtained when considering the “Type 2-high” phenotype, supporting the view that these two biomarkers are not interchangeable and that the use of both biomarkers in combination may allow for better targeted and personalized treatment for at least certain subsets of asthma patients [7, 10, 11].

The more prevalent combinations of phenotypes observed in this study were AwObesity together with either B-Eos-high or B-Eos&FeNO-low phenotypes. This supports the view that obesity-related asthma, despite often suggested to be a separate asthma phenotype associated with non-eosinophilic airway inflammation [9, 55, 56], may also be associated to eosinophilic inflammation.

Given the high prevalence in the US population, in this sample, obesity is likely to be a comorbidity, rather than the primary reason for asthma [57]; however, we defined the AwObesity phenotype as a separate group, since the interdependence on inflammatory markers to targeting different asthma therapies makes essential the accurate characterization of inflammation in obese asthmatic subjects [19, 58]. In addition, the relevance of defining the AwObesity phenotype is supported by the data as the weighted proportion of overlap is similar when excluding AwObesity or B-Eos-high phenotype from the analysis (data not shown). Moreover, the weighted proportion of subjects with “non-classified” asthma doubled when excluding the AwObesity phenotype (increasing to 32% in the < 40 years-old and 29% in ≥ 40 years old).

Having multiple asthma phenotypes was more common in older subjects and in non- and former- smokers; whether this is due to a general increase of comorbidities with age [58–60] and/or an interaction with environmental factors [61] cannot be specifically addressed by this study design. Nevertheless, when interpreting these results one should bear in mind that AwCOPD is associated to older age and prior/current smoking while FeNO increases with age and decreases with smoking [62, 63].

Interestingly, subjects with a higher number of concomitant asthma phenotypes presented reduced lung function and this association remained when controlling for potential confounders by multiple regression analysis. This shows that having multiple phenotypes is independently associated with reduced lung function, suggesting a cumulative effect of different disease processes.

Moreover, patients having several commonly reported asthma phenotypes had higher odds of using more controller medications, supporting the view that these patients are those with more complex disease and higher asthma morbidity [9, 15]. This also suggests that these asthma patients have an inadequate response to prescribed therapies, since lung function was reduced, and that they may represent a group of patients with the need for add-on treatment. However, the choice of specific treatments, such as biological therapies, will be more difficult considering the complexity introduced by having multiple phenotypes.

The lack of significant associations between multiple phenotypes and the other asthma-related outcomes may be difficult to understand. A possible explanation could be that the prescribed medication is effective against asthma symptoms and attacks but less effective against (subclinical) processes that cause long-term reduction in lung function. However, the results could also be related to data collection methods, as lung function measurement and the way medication use was ascertained, were less dependent on patient recall than the self-reported variables that were used for the outcomes with null results in the present study. Further studies should be done, adding the quantitative assessment of asthma attacks/asthma-related ED visits, and also including the age of asthma onset, that could have an influence on asthma-related outcomes.

This study has several limitations. First, because of its cross-sectional design, it was not possible to evaluate interactions between phenotypes over time in patients with concomitant phenotypes, nor was it possible to determine which phenotype occurred first. Second, although there were differences between the included and excluded groups in the variables age, BMI, non-Hispanic white/black subjects, having finished high school and OCS use, the majority were not used for phenotypic classification and did not affect the outcomes, as shown in sensitivity analysis. Third, as our asthma and COPD definitions were based on self-reported diagnosis, rather than relying on lung function tests, the acquired information is subject to recall bias and misclassification. However, these definitions have been commonly used in NHANES reports [7, 11, 15] and have proven to be reasonably reliable [64, 65]. Moreover, we have used the most frequent combination of questions seen in epidemiological studies [65, 66], and we also included questions on recent wheeze and/or asthma attacks, which should reduce the risk of including individuals without true disease. Also, we stratified the analysis by age (at 40 years), as used in other COPD studies [37, 67], in order to improve the clinical value into the interpretation of phenotyping data, as the overlap among asthma phenotypes will be different among those less than age 40 and those older than 40 (with higher possibility of having COPD) [37]. Fourth, the lack of other biomarkers in the present NHANES years, prevented the analysis of other asthma phenotypes and the use of alternative definitions. However, we analyzed biomarkers of type 2-inflammation in both blood and exhaled air that previously have been shown to independently relate to asthma morbidity [7, 11]. Fifth, as there is no consensual definition of biomarker-defined asthma phenotypes, we based our definitions on cut-offs used in previous studies to discriminate patients in single asthma phenotypes [32–35], rather than on any reported specificity or sensitivity for predicting asthma morbidity or response to therapeutics [68, 69]. For high probability of eosinophilic inflammation, the cut-off value for FeNO has been suggested to be > 50 ppb for adults [70]. However, we chose a FeNO cut-off of 35 ppb, based on the mean baseline FeNO levels of patients included in randomized controlled trials of anti-IL-13 treatment [33, 69]. In spite of using this lower cut-off, 77 subjects with current asthma could not be classified as having any of the predefined phenotypes, indicating the need for better, and probably personalized, cut-offs for biomarkers in asthma.

Furthermore, we could not demonstrate a clear effect of ongoing controller medication in the phenotypic classification in our data. No significant associations were observed, probably at least partially explained with the exclusion of the participants with ICS/OCS use < 48 h prior to the exhaled NO measurements. Also, contrary to the expected, we observed a higher proportion of patients treated with ICS/OCS within both B-Eos-high and FeNO-high phenotypes than the B-Eos&FeNO-low phenotype. A plausible explanation is that subjects with ongoing inflammation have more clear asthma, with more symptoms, and, thus, a higher need of treatment. B-Eos&FeNO-low asthma is a heterogeneous group with less need of controller treatment, and because the treatment is ineffective, it may be that medication use and even prescription has been stopped.

Finally, even though we did not specifically analyze the overlap of asthma phenotypes in patients with severe asthma, the significant association between presenting more than one phenotype and being treated with multiple asthma controller medications suggests higher asthma severity in this subset [71]. Also, we did not consider individual environmental factors, such as air pollution and/or indoor allergens, that could influence asthma phenotypes. Further studies describing the overlap in patients with severe asthma and studies examining asthma patients exposed to different environmental factors, such as subjects who live in cities versus in rural areas are needed.

This study indicates that the overlap of commonly reported asthma phenotypes is observed also in non-selected asthma patients from the general population. Our findings highlight the importance of classifying asthma patients with regard to applicable phenotypes, rather than using a single asthma phenotype, to enable the development of adequate targeted strategies to avoid lung function impairment. However, further data is required, such as that from higher order analysis, using data-mining methods possibly combined with those that rely on predefined hypotheses. This synergy is expected to improve the knowledge on asthma phenotypes and, ultimately, to lead to more personalized treatment strategies [53, 54].

## Conclusions

In conclusion, a prevalent overlap of commonly reported phenotypes was observed in asthma patients identified from the general population. Subjects classified as having multiple phenotypes used more controller medications and had reduced lung function. Thus, the complexity and unique features of concomitant asthma phenotypes may require a broader data analysis approach, based on a combination of clinical information and biomarkers resulting in better characterization of patients. This could lead to better asthma outcomes, particularly preserved lung function.

## Additional files

**Additional file 1.** Supplementary Methods.

**Additional file 2: Table S1.** Distribution and comparisons between the FeNO and B-Eos cut-offs used in this study, among individuals with current asthma.

**Additional file 3: Table S2.** Weighted percentages and comparisons of asthma-related outcomes among subjects with a single asthma phenotype versus: non-classified, and specific combinations of asthma phenotypes.

**Additional file 4: Table S3.** Weighted percentages and comparisons of asthma-related outcomes among subjects with a single asthma phenotype versus: non-classified, and specific combinations of asthma phenotypes.

**Additional file 5: Table S4.** Multivariable logistic regression models between each asthma-related outcome and having multiple asthma phenotypes, adjusted for co-variables.

**Additional file 6: Fig. S1.** Proportions (weighted to the US population) of subjects taking asthma controller medications stratified into the different phenotypes, among all participants included for asthma phenotype classification (left) and only in those with a single phenotype (right). P-values <0.05 were indicated. NA: Non-applicable (not possible to determine because some participants had both B-Eos-high and FeNO-high asthma phenotypes).

## Abbreviations

aOR: adjusted odds ratios
ATS/ERS: American Thoracic Society/European Respiratory Society
AwCOPD: asthma with concurrent COPD
AwObesity: asthma with obesity
B-Eos: blood eosinophils
BMI: body mass index
COPD: chronic obstructive pulmonary disease
CI: confidence intervals
ED: emergency department
FeNO: fraction of exhaled nitric oxide
FEV_1_: forced expiratory volume in 1 s
FEV_1_/FVC: ratio of forced expiratory volume in 1 s to forced vital capacity
ICS: inhaled corticosteroids
IgE: immunoglobulin E
IL: interleukin
LLN: lower limit of normal
NHANES: National Health and Nutrition Examination Survey (USA)
OCS: oral corticosteroids
Q1: first quartile
Q3: third quartile
Th2: T helper cell type 2
