# Supplementary material for: Having concomitant asthma phenotypes is common and independently relates to poor lung function in NHANES 2007–2012
Source: Clin Transl Allergy. 2018 May 4;8:13. doi: 10.1186/s13601-018-0201-3 (PMC5934840; doi:10.1186/s13601-018-0201-3)
Supplement: Supplementary file 1 — Additional file 1. Supplementary Methods. [file 13601_2018_201_MOESM1_ESM.docx]

**ONLINE SUPPLEMENT**

**Having concomitant asthma phenotypes is common and independently relates to poor lung function in NHANES 2007-2012**

Rita Amaral, MSc.^1,2^, João A. Fonseca, M.D., Ph.D.^1,3,4^, Tiago Jacinto, Ph.D.^1,2,4^, Ana M. Pereira, M.D.^1,4^, Andrei Malinovschi, M.D., Ph.D.^5^, Christer Janson, M.D., Ph.D.^6^, Kjell Alving, M.D., Ph.D.^7^

^1^CINTESIS- Center for Health Technology and Services Research, Faculty of Medicine, University of Porto, Portugal;

^2^Dept. of Cardiovascular and Respiratory Sciences, Porto Health School, Porto, Portugal

^3^MEDCIDS- Dept. of Community Medicine, Information, and Health Sciences: Faculty of Medicine, University of Porto, Portugal;

^4^Dept. of Allergy: Instituto & Hospital CUF, Porto, Portugal;

^5^Dept. of Medical Sciences: Clinical Physiology, Uppsala University, Uppsala, Sweden;

^6^Dept. of Medical Sciences: Respiratory Medicine and Allergology, Uppsala University, Uppsala, Sweden;

^7^Dept. of Women’s and Children’s Health: Paediatric Research, Uppsala University, Uppsala, Sweden

# Supplementary methods

## Study design

The National Health and Nutrition Examination Survey (NHANES) is a program of studies designed to assess the health and nutritional status of adults and children in the United States. The survey is unique in that it combines interviews and physical examinations.

The NHANES program began in the early 1960s and has been conducted as a series of surveys focusing on different population groups or health topics. The survey examines a nationally representative sample of about 5,000 persons each year. These persons are located in counties across the country, 15 of which are visited each year, to produce reliable statistics, NHANES over-samples persons 60 and older, African Americans, and Hispanics.

The NHANES interview includes demographic, socioeconomic, dietary, and health-related questions. The examination component consists of medical, dental, and physiological measurements, as well as laboratory tests administered by highly trained medical personnel.

All participants visit the physician. Dietary interviews and body measurements are included for everyone. All but the very young have a blood sample taken and will have a dental screening.

Health interviews are conducted in respondents’ homes. Health measurements are performed in specially-designed and equipped mobile centers, which travel to locations throughout the country. An advanced computer system using high-end servers, desktop PCs, and wide-area networking collect and process all of the NHANES data. Touch-sensitive computer screens let respondents enter their own responses to certain sensitive questions in complete privacy.

Further details on survey design and respective survey questionnaires can be found in http://www.cdc.gov/nchs/nhanes/about_nhanes.htm.

## Variables

Demographic characteristics, such as age, gender, body mass index (BMI), race/ethnicity, and educational status were analyzed:

- *Body mass index (BMI)* was calculated and classified based on the WHO definition (S1): underweight (BMI ≤18.4 kg/m^2^); normal (18.5-24.9 kg/m^2^), overweight (25–29.9 kg/m^2^), and obese (≥30 kg/m^2^);
- *Educational status* was divided in: less than high school (< high school) and completion or greater than high school (≥high school).

*Current smoking* was considered if participants had a positive answer to both questions: “Have you smoked ≥100 cigarettes during lifetime” and “Do you now smoke cigarettes?”. If participants answered positively to the first question but negatively to the second one, they were considered ex-smokers.

*Blood eosinophils (B-Eos)* were part of the complete blood counts and assessed on a Beckman Coulter MAXM® instrument (Beckman Coulter, Fullerton, Calif). Blood samples were collected at the NHANES Mobile Examination Center. A detailed description of the laboratory method used can be found elsewhere (S2).

*FeNO* measurements were also performed at the mobile examination center using the analyzer NIOX MINO® (Aerocrine, Solna, Sweden) (S3) and valid measurements were defined in accordance with ATS/ERS guidelines (S4). *Spirometric* procedures followed the ATS/ERS recommendations (S5), and a minimum of 3 acceptable and reproducible measurements were obtained (S6). After predicted values of basal FEV1 and FEV1/FVC were calculated according to Hankinson et al (S7) with an ethnicity correction (S8), abnormal lung function was defined according to the lower limit of normal (LLN), which classifies subjects with spirometry values below the lower fifth percentile of an aged-matched healthy reference group as abnormal (S9). FeNO and spirometric measurements not fulfilling ATS/ERS recommendations (S4,S5) were excluded (n=653).

*Self-reported asthma attacks* and *asthma-related emergency department (ED) visits* (Yes/No), in the past 12 months were analyzed.

*Work/school absenteeism* was defined as having at least one day lost at work/school due to wheezing (Yes/No).

*Asthma symptoms* were evaluated with the following questions regarding the last 12 months: “Had wheezing/whistling in your chest?”; “Had disturbed sleep due to wheezing?”; “Had dry hard cough at night, not associated with a cold for at least 14 days in a row?”; “Had wheezing during/after exercise?”; and “Had limited activity due to wheezing?”.

*Self-reported rhinitis* was defined by an affirmative answer to “During the past 12 months, have you had an episode of hay fever?”.

*Use of reliever/rescue medication* for asthma was considered if the participant used short-acting β_2_-agonist, anticholinergic, or inhaled corticosteroids (ICS)/formoterol.

*Controller medication* included: ICS; leukotriene modifiers; long-acting inhaled β_2_-agonist (LABA) and ICS combination; ICS/LABA associated with methylxanthine, cromoglycate, and/or oral corticosteroids. Details on the prescription medication data collection in NHANES 1999–2012 are available elsewhere (S10).

## Statistical analysis

A multivariate logistic regression was performed in order to explore the association of concomitant (having at least 2 concurrent) phenotypes with each asthma-related outcomes. Separate models were run using each asthma-related outcome (asthma attack, asthma-related ED visit, ≥2 asthma symptoms, work/school absenteeism, rescue medication, and ≥2 controller medication and abnormal lung function) as dependent variable and having multiple phenotypes as independent variables.

Model fit was assessed using the *svylogitgof* function for complex survey data (S11).

To create the Venn-Euler diagram, we excluded subjects with incomplete information for at least one of the following: self-reported medical conditions, BMI, smoking history, and FeNO or B-Eos count (listwise deletion of missing data) (S12).

# References of supplementary methods

S1. WHO. Physical status: the use and interpretation of anthropometry. Report of a WHO Expert Committee. *World Health Organization technical report series* 1995;1–452.

S2. CDC; National Health and Nutrition Examination 2011-2012. Complete Blood Count [updated 2013; accessed 2017 Aug]. Available from: <http://www.cdc.gov/nchs/data/nhanes/nhanes_11_12/cbc_g_met_he.pdf>

S3. CDC; National Health and Nutrition Examination Survey. Respiratory Health ENO Procedures Manual [updated 2011; accessed 2017 Aug]. Available from: [www.cdc.gov/nchs/data/nhanes/nhanes_11_12/Respiratory_Health_ENO_Procedures_Manual.pdf](http://www.cdc.gov/nchs/data/nhanes/nhanes_11_12/Respiratory_Health_ENO_Procedures_Manual.pdf)

S4. ATS/ERS. ATS/ERS Recommendations for Standardized Procedures for the Online and Offline Measurement of Exhaled Lower Respiratory Nitric Oxide and Nasal Nitric Oxide. *Am J Respir Crit Care Med* 2005;**171**(8):912–3

S5. Miller MR. Standardisation of spirometry. *Eur Respir J* 2005;**26**(2):319–38.

S6. CDC; National Health and Nutrition Examination Survey. Respiratory Health Spirometry Procedures Manual [updated 2011; accessed 2017 Aug]. Available from: <https://www.cdc.gov/nchs/data/nhanes/nhanes_11_12/spirometry_procedures_manual.pdf>

S7. Hankinson JL, Odencrantz JR, Fedan KB. Spirometric Reference Values from a Sample of the General U.S. Population. *Am J Respir Crit Care Med* 199;**159**(1):179–87.

S8. Hankinson JL, Kawut SM, Shahar E, Smith LJ, Stukovsky KH, Barr RG. Performance of American Thoracic Society-Recommended Spirometry Reference Values in a Multiethnic Sample of Adults. *Chest* 2010;**137**(1):138–45.

S9. Pellegrino R, Viegi G, Brusasco V, Crapo RO, Burgos F, Casaburi R, et al. Interpretative strategies for lung function tests. *Eur Respir J* 2005;1;**26**(5):948–68.

S10. CDC; National Health and Nutrition Examination Survey 1988-2012. Prescription Medications - Drug Information [updated 2014; accessed 2017 Aug]. Available from: <http://wwwn.cdc.gov/Nchs/Nhanes/1999-2000/RXQ_DRUG.htm>

S11. Archer K, Lemeshow S. Goodness-of-fit test for a logistic regression model fitted using survey sample data. *Stata J* 2006;**6**:97–105.

S12. Peugh JL, Enders CK. Missing data in educational research: A review of reporting practices and suggestions for improvement. *Review of Educational Research* 2004;**74**;525-55.
