# Supplementary material for: Having concomitant asthma phenotypes is common and independently relates to poor lung function in NHANES 2007–2012
Source: Clin Transl Allergy. 2018 May 4;8:13. doi: 10.1186/s13601-018-0201-3 (PMC5934840; doi:10.1186/s13601-018-0201-3)
Supplement: Supplementary file 2 — Additional file 2: Table S1. Distribution and comparisons between the FeNO and B-Eos cut-offs used in this study, among individuals with current asthma. [file 13601_2018_201_MOESM2_ESM.docx]

## Additional file 2: Table S1. Distribution and comparisons between the FeNO and B-Eos cut-offs used in this study, among individuals with current asthma

|  |  | **Total**  n (weigthed %) | **B-Eos**  n (weigthed %) | | |  | **p-value** | | |
| --- | --- | --- | --- | --- | --- | --- | --- | --- | --- |
|  |  |  | **Class I**  <150/mm^3^ | **Class II**  150-300/mm^3^ | **Class III**  ≥300/mm^3^ |  | I vs II | II vs III | I vs III |
| **Current asthma (n= 1,059)** | | | 332 (37) | 276 (27) | 348 (36) |  |  |  |  |
| **FeNO** | <20ppb | 501 (65) | 184 (73) | 146 (67) | 141 (50) |  | 0.12 | **<0.001** | **<0.001** |
|  | ≥20ppb | 273 (35) | 60 (27) | 66 (33) | 130 (50) |  |  |  |  |
|  | <35ppb | 636 (82) | 229 (94) | 186 (86) | 184 (65) |  | **0.02** | **<0.001** | **<0.001** |
|  | ≥35ppb | 138 (18) | 15 (6) | 26 (14) | 87 (35) |  |  |  |  |

FeNO: Fraction of exhaled nitric oxide; B-Eos: blood eosinophils.

P-values <0.05 are presented.
