# Supplementary material for: Having concomitant asthma phenotypes is common and independently relates to poor lung function in NHANES 2007–2012
Source: Clin Transl Allergy. 2018 May 4;8:13. doi: 10.1186/s13601-018-0201-3 (PMC5934840; doi:10.1186/s13601-018-0201-3)
Supplement: Supplementary file 3 — Additional file 3: Table S2. Weighted percentages and comparisons of asthma-related outcomes among subjects with a single asthma phenotype versus: non-classified, and specific combinations of asthma phenotypes. [file 13601_2018_201_MOESM3_ESM.docx]

## Additional file 3: Table S2. Weighted percentages and comparisons of asthma-related outcomes among subjects with a single asthma phenotype versus: non-classified, and specific combinations of asthma phenotypes.

| **Weighted %** | **Total** | | **Asthma attack** | **Asthma-related ED** | **≥2 asthma symptoms** | **Work/school absenteeism** | **Asthma medication** | |  | **Lung function** | | |
| --- | --- | --- | --- | --- | --- | --- | --- | --- | --- | --- | --- | --- |
|  |  |  |  |  |  |  | ≥1 reliever medication | ≥2 controller medication |  | FEV_1_ <LLN | FEV_1_% predicted§ | FEV_1_/FVC <LLN |
| **Single phenotypes*** | 43 | | 67 | 21 | 59 | 17 | 38 | 14 |  | 14 | 94  (86-97) | 23 |
| AwObesity | 38 | | 61 | 27 | 59 | 15 | 31 | 14 |  | 18 | 92  (84-104) | 16 |
| B-Eos-high | 22 | | 81 | 29 | 70 | 19 | 47 | 15 |  | 17 | 95  (84-99) | 27 |
| FeNO-high | 8 | | 80 | 6 | 31 | 3 | 33 | 28 |  | 11 | 94  (87-96) | 55 |
| B-Eos&FeNO-low | 30 | | 64 | 14 | 55 | 20 | 46 | 9 |  | 7 | 93  (88-102) | 20 |
| AwCOPD | 2 | | 24 | 20 | 93 | 61 | 6 | 23 |  | 30 | 97  (57-97) | 30 |
| **Non-classified†** | 14 | | 75 | **8** | 59 | 14 | 36 | 15 |  | 25 | 90  (80-103) | 23 |
| **Multiple phenotypes‡** | | 43 | 72 | 23 | 64 | 14 | **49** | **27** |  | **34** | **83**  **(74-95)** | 34 |
| AwObesity + others | 48 | | 71 | 23 | 65 | 16 | 48 | **25** |  | **30** | **81**  **(74-92)** | 26 |
| B-Eos-high + others | 19 | | 75 | 20 | 65 | 14 | **51** | **31** |  | **39** | **80**  **(69-91)** | **42** |
| FeNO-high + others | 10 | | 68 | 16 | 66 | 17 | 51 | **30** |  | **37** | **81**  **(74-92)** | 38 |
| B-Eos&FeNO-low + others | 9 | | 78 | 33 | 59 | 18 | 42 | 18 |  | 25 | 90  (74-100) | 21 |
| AwCOPD + others | 5 | | 61 | 22 | 61 | 20 | **66** | **38** |  | **50** | **74**  **(63-86)** | **63** |
| **Specific combinations of phenotypes** | | | |  |  |  |  |  |  |  |  |  |
| B-Eos-high + AwObesity | 8 | | 77 | 25 | 62 | **8** | 46 | 27 |  | **32** | 85  (77-95) | 32 |
| AwObesity+ B-Eos&FeNO-low | 7 | | 79 | 30 | 64 | 13 | 35 | 16 |  | 18 | 94  (77-101) | 9 |
| B-Eos-high + FeNO-high | 5 | | 73 | 19 | 66 | **6** | 44 | **35** |  | 41 | 80  (69-90) | **51** |
| B-Eos-high + FeNO-high + AwObesity | 3 | | 80 | 16 | 65 | 40 | **67** | 22 |  | **33** | **79**  **(75-86)** | 30 |
| FeNO-high + AwObesity | 2 | | **26** | 8 | 67 | 12 | 47 | 27 |  | 32 | 93  (74-96) | 9 |
| B-Eos-high + AwCOPD | 1 | | 71 | 14 | 73 | 25 | 69 | 4 |  | 22 | 110  (90-110) | 43 |
| AwObesity + AwCOPD | 1 | | **20** | 19 | 67 | 0 | 71 | 12 |  | 14 | **85**  **(83-85)** | 14 |
| AwCOPD+ B-Eos&FeNO-low | 1 | | 76 | 44 | **20** | 12 | **84** | 44 |  | **71** | **66**  **(63-77)** | **85** |

AwObesity: Asthma with obesity; AwCOPD: Asthma with COPD; ED: Emergency-department; FEV_1_: Forced expiratory volume in 1 second; FEV_1_/FVC: Forced expiratory volume in 1 second and functional vital capacity ratio; LLN: Lower limit of normality.

Values are presented as weighted percentages and significant associations (p<0.05) between multiple and single phenotypes are presented in bold.

*Subjects having only one of the 5 asthma phenotypes: AwObesity, B-Eos-high, FeNO-high, B-Eos&FeNO-low or AwCOPD. **†** Subjects with non-single and non-multiple phenotypes. **‡**Subjects having at least one of the other asthma phenotypes. § Presented as median (Q1-Q3).
