# Supplementary material for: Having concomitant asthma phenotypes is common and independently relates to poor lung function in NHANES 2007–2012
Source: Clin Transl Allergy. 2018 May 4;8:13. doi: 10.1186/s13601-018-0201-3 (PMC5934840; doi:10.1186/s13601-018-0201-3)
Supplement: Supplementary file 4 — Additional file 4: Table S3. Weighted percentages and comparisons of asthma-related outcomes among subjects with a single asthma phenotype versus: non-classified, and specific combinations of asthma phenotypes. [file 13601_2018_201_MOESM4_ESM.docx]

## Additional file 4: Table S3. Weighted percentages and comparisons of asthma-related outcomes among subjects with a single asthma phenotype versus: non-classified, and specific combinations of asthma phenotypes.

| **Weighted %** | **Total** | **Asthma attack** | **Asthma-related ED** | **≥2 asthma symptoms** | **Work/school absenteeism** | **Asthma medication** | |  | **Lung function** | | |
| --- | --- | --- | --- | --- | --- | --- | --- | --- | --- | --- | --- |
|  |  |  |  |  |  | ≥1 reliever medication | ≥2 controller medication |  | FEV_1_ <LLN | FEV_1_% predicted§ | FEV_1_/FVC <LLN |
| **Single phenotypes** ***** | 49 | 68 | 22 | 59 | 15 | 40 | 18 |  | 18 | 92  (82-99) | 28 |
| AwObesity | 31 | 61 | 27 | 59 | 15 | 29 | 15 |  | 18 | 92  (84-111) | 16 |
| Type 2-high**†** | 43 | 77 | 23 | 59 | 11 | 46 | 25 |  | 25 | 90  (80-98) | 41 |
| B-Eos&FeNO-low | 24 | 64 | 14 | 55 | 20 | 45 | 9 |  | 7 | 93  (88-102) | 20 |
| AwCOPD | 2 | 24 | 20 | 93 | 61 | 11 | 24 |  | 20 | 97  (57-97) | 30 |
| **Non-classified‡** | 13 | 75 | **8** | 59 | 14 | 36 | 15 |  | 25 | 90  (80-103) | 23 |
| **Multiple phenotypes** **\|\|** | 38 | 71 | 24 | 65 | 19 | **51** | **27** |  | **32** | **85**  **(74-96)** | 30 |
| AwObesity+ others | 26 | 71 | 23 | 67 | 20 | 49 | 26 |  | **30** | **85**  **(75-96)** | 26 |
| Type 2-high+ others | 19 | 70 | 20 | 68 | 21 | **54** | **32** |  | **37** | **82**  **(74-93)** | 36 |
| B-Eos&FeNO-low+ others | 9 | 78 | 33 | 59 | 18 | 42 | 18 |  | 25 | 90  (74-100) | 21 |
| AwCOPD+ others | 6 | 64 | 23 | 66 | 18 | **66** | **43** |  | **50** | **74**  **(63-86)** | **63** |
| **Specific combinations of phenotypes** | | |  |  |  |  |  |  |  |  |  |
| Type 2-high+ AwObesity | 15 | 70 | 22 | 66 | 22 | 52 | 25 |  | **32** | **83**  **(76-94)** | 28 |
| Type 2-high+ AwCOPD | 2 | 74 | 24 | **86** | 11 | **69** | **47** |  | 18 | 90  (81-110) | **52** |

AwObesity: Asthma with obesity; AwCOPD: Asthma with COPD; ED: Emergency-department; FEV_1_: Forced expiratory volume in 1 second; FEV_1_/FVC: Forced expiratory volume in 1 second and functional vital capacity ratio; LLN: Lower limit of normality.

Values are presented as weighted percentages and significant associations (p<0.05) between multiple and single phenotypes are presented in bold.

*Subjects with only one of the 4 asthma phenotypes: AwObesity, Type 2-high, B-Eos&FeNO-low or AwCOPD. **†**Type 2-high (B-Eos-high or FeNO-high), **‡** subjects with non-single and non-multiple phenotypes. ||Subjects having at least one of the other asthma phenotypes. § Presented as median (Q1-Q3)
