# Supplementary material for: Having concomitant asthma phenotypes is common and independently relates to poor lung function in NHANES 2007–2012
Source: Clin Transl Allergy. 2018 May 4;8:13. doi: 10.1186/s13601-018-0201-3 (PMC5934840; doi:10.1186/s13601-018-0201-3)
Supplement: Supplementary file 5 — Additional file 5: Table S4. Multivariable logistic regression models between each asthma-related outcome and having multiple asthma phenotypes, adjusted for co-variables. [file 13601_2018_201_MOESM5_ESM.docx]

# Additional file 5: Table S4. Multivariable logistic regression models between each asthma-related outcome and having multiple asthma phenotypes, adjusted for co-variables.

|  | **Asthma attack**  aOR (95% CI) | **Asthma-related ED**  aOR (95% CI) | **≥2 asthma symptoms**  aOR (95% CI) | **Work/school absenteeism** aOR (95% CI) | **≥1 reliever medication***  aOR (95% CI) | **FEV_1_/FVC <LLN**  aOR (95% CI) |
| --- | --- | --- | --- | --- | --- | --- |
| **Multiple vs. single phenotype** | 1.27 (0.78-2.06) | 1.17 (0.74-1.86) | 1.26 (0.76-2.11) | 0.79 (0.37-1.68) | 1.55 (0.97-2.49) | 1.74 (0.94-3.24) |
| **Female** | 1.34 (0.87-2.07) | **2.05 (1.08-3.90)** | **1.96 (1.16-3.31)** | 1.35 (0.66-2.79) | 0.88 (0.53-1.49) | **0.53 (0.31-0.90)** |
| **Age ≥ 40 yrs** | 0.80 (0.50-1.27) | 0.91 (0.59-1.41) | 0.94 (0.55-1.60) | 1.09 (0.60-2.00) | 1.08 (0.67-1.73) | 1.50 (0.90-2.49) |
| **Caucasian vs. other** | 0.89 (0.54-1.46) | **0.38 (0.23-0.64)** | 0.72 (0.46-1.15) | 0.76 (0.39-1.50) | 1.03 (0.65-1.63) | 0.92 (1.16-3.51) |
| **Current smoker** **vs. non-/ex-smokers** | 0.89 (0.55-1.43) | 1.60 (0.94-2.72) | 1.22 (0.69-2.14) | 0.82 (0.37-1.83) | **1.95 (1.35-2.83)** | **2.02 (1.16-3.51)** |
| **Rhinitis** | 0.96 (0.59-1.57) | 0.77 (0.38-1.56) | 0.88 (0.50-1.52) | 0.70 (0.32-1.53) | 0.66 (0.42-1.04) | 1.04 (0.62-1.75) |
| **Goodness-of-fit test** | |  |  |  |  |  |
| χ^2^ (p-value) | 0.59 (0.80) | 0.59 (0.81) | 1.33(0.22) | 1.27 (0.25) | 1.14 (0.33) | 3.05 (0.002) |

CI: confidence interval; aOR: adjusted odds ratio; ED: emergency-department; FEV_1_/FVC: Forced expiratory volume in one second and forced vital capacity ratio; LLN: Lower Limit of Normal; χ^2^: Chi-square.

All asthma-related outcomes were analyzed separately and treated as dependent variables. The aOR values with p<0.05 are presented in bold.

*Short-acting β_2_-agonist and/or anticholinergic.
