# Supplementary material for: Having concomitant asthma phenotypes is common and independently relates to poor lung function in NHANES 2007–2012
Source: Clin Transl Allergy. 2018 May 4;8:13. doi: 10.1186/s13601-018-0201-3 (PMC5934840; doi:10.1186/s13601-018-0201-3)
Supplement: Supplementary file 6 — Additional file 6: Fig. S1. Proportions (weighted to the US population) of subjects taking asthma controller medications stratified into the different phenotypes, among all participants included for asthma phenotype classification (left) and only in those with a single phenotype (right). P-values <0.05 were indicated. NA: Non-applicable (not possible to determine because some participants had both B-Eos-high and FeNO-high asthma phenotypes). [file 13601_2018_201_MOESM6_ESM.pdf]

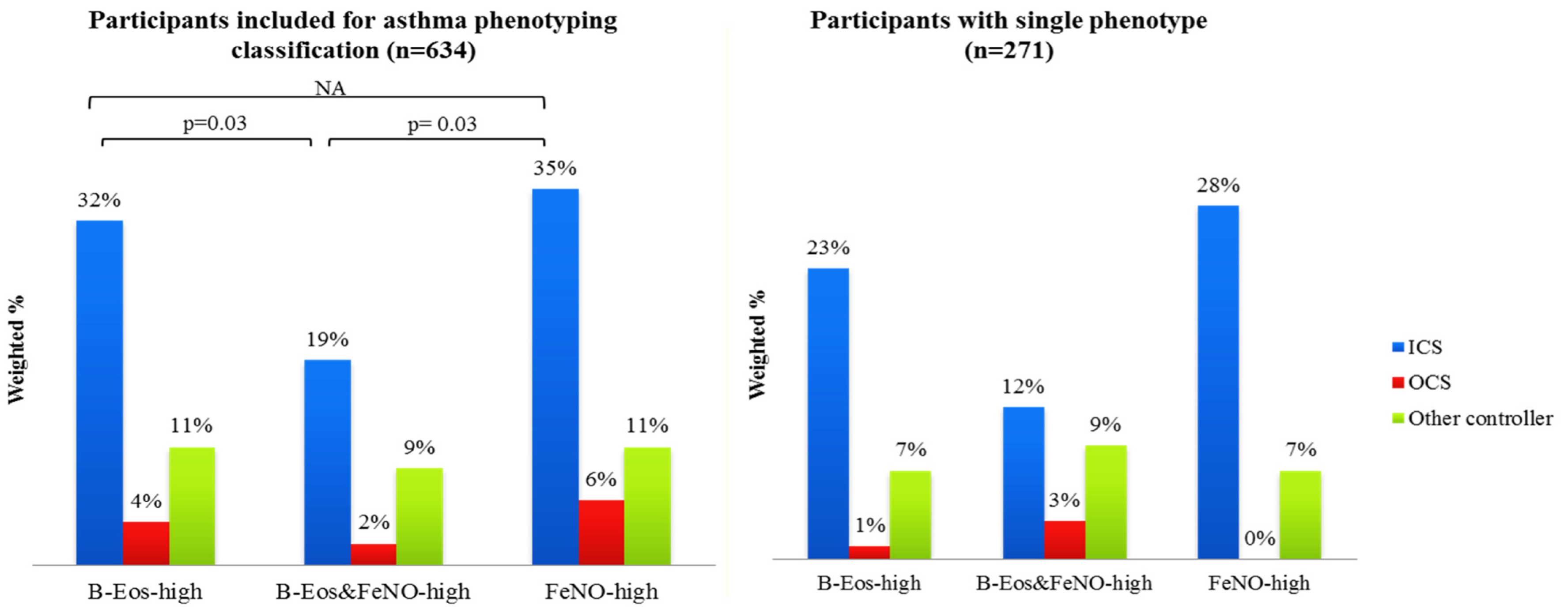

Figure S1: Proportions (weighted to the US population) of subjects taking asthma controller medications stratified into the different phenotypes, among all participants included for asthma phenotype classification (left) and only in those with a single phenotype (right). P-values <0.05 were indicated. NA: Non-applicable (not possible to determine because some participants had both B-Eos-high and FeNO-high asthma phenotypes).
